# Transcriptomic and splicing changes underlying tomato responses to combined water and nutrient stress

**DOI:** 10.3389/fpls.2022.974048

**Published:** 2022-11-25

**Authors:** Alessandra Ruggiero, Paola Punzo, Michael James Van Oosten, Valerio Cirillo, Salvatore Esposito, Antonello Costa, Albino Maggio, Stefania Grillo, Giorgia Batelli

**Affiliations:** ^1^ CNR-IBBR, National Research Council of Italy, Institute of Biosciences and Bioresources, Research Division, Portici, Italy; ^2^ Department of Agricultural Sciences, University of Naples, Federico II, Portici, Italy; ^3^ CREA-CI, Council for Agricultural Research and Economics, Research Centre for Cereal and Industrial Crops, Foggia, Italy

**Keywords:** differential gene expression, differential alternative splicing, leaf, root, water deficit, low nitrate, tolerant and sensitive genotypes

## Abstract

Tomato is a horticultural crop of high economic and nutritional value. Suboptimal environmental conditions, such as limited water and nutrient availability, cause severe yield reductions. Thus, selection of genotypes requiring lower inputs is a goal for the tomato breeding sector. We screened 10 tomato varieties exposed to water deficit, low nitrate or a combination of both. Biometric, physiological and molecular analyses revealed different stress responses among genotypes, identifying T270 as severely affected, and T250 as tolerant to the stresses applied. Investigation of transcriptome changes caused by combined stress in roots and leaves of these two genotypes yielded a low number of differentially expressed genes (DEGs) in T250 compared to T270, suggesting that T250 tailors changes in gene expression to efficiently respond to combined stress. By contrast, the susceptible tomato activated approximately one thousand and two thousand genes in leaves and roots respectively, indicating a more generalized stress response in this genotype. In particular, developmental and stress-related genes were differentially expressed, such as hormone responsive factors and transcription factors. Analysis of differential alternative splicing (DAS) events showed that combined stress greatly affects the splicing landscape in both genotypes, highlighting the important role of AS in stress response mechanisms. In particular, several stress and growth-related genes as well as transcription and splicing factors were differentially spliced in both tissues. Taken together, these results reveal important insights into the transcriptional and post-transcriptional mechanisms regulating tomato adaptation to growth under reduced water and nitrogen inputs.

## Introduction

Agriculture is the single human activity consuming the highest amount of water. An increased frequency and duration of drought spells, due to climate change dynamics, may result in a higher water demand for agricultural activities. Nitrate fertilization causes the formation of greenhouse gases, contributing to climate change (Voigt et al., 2017). Thus, the development of genotypes with lower demands in terms of water and nitrate inputs, coupled with optimized management practices, may reduce the environmental impact of agricultural practices while improving stress resilience, and is thus considered a priority in plant breeding.

Tomato is an important vegetable crop in terms of economic value and as a dietary source of beneficial compounds. Cultivation in open field is often challenged by drought stress, which, depending on duration and intensity, can cause stomata closure and reduced photosynthesis, a reduced leaf water content and an overall reduction in size and leaf number, leading to a lower leaf area and biomass accumulation (Iovieno et al., 2016; Landi et al., 2017; Zhou et al., 2017). In addition, drought can reduce nitrate uptake in tomato, imposing further constraints on plant growth (Sánchez-Rodríguez et al., 2011).

Similarly, nitrogen deficiency also has macroscopic effects on plant growth and accumulation of dry matter, by causing reduced protein synthesis and hormone imbalances, among other effects (Scholberg et al., 2000; Scheible et al., 2004).

Drought and N availability are perceived by root systems, leading to modifications of root architecture, with tomato primary root growth favored over lateral root development under water deficit, and a general inhibition of root growth in the case of low N (Abenavoli et al., 2016; Machado et al., 2022).

Genome-wide expression studies, in addition to classical genetics have contributed to identify drought responsive genes and QTLs that may be important to improve tomato tolerance to drought stress (Gur and Zamir, 2004; Gong et al., 2010; Iovieno et al., 2016; Liu et al., 2017). Transporters and enzymes regulated by nitrate availability and important for uptake, translocation and metabolism of nitrate have been identified (Abenavoli et al., 2016). However, a combination of drought stress and nitrate deficiency, and the resulting molecular changes in tomato has been little explored.

Alternative splicing (AS) is a co- and post-transcriptional regulatory mechanism that gives rise to multiple transcripts from a single gene. AS occurs when the spliceosome recognizes different splice sites in the pre-mRNA, leading to inclusion of introns (Intron Retention, IR) or exclusion of exons (Exon Skipping, ES), completely or partially (Alternative Donor or Acceptor site, A5’SS or A3’SS), in mature mRNA (Syed et al., 2012; Chaudhary et al., 2019a).

In plants, AS regulates up to 70% of multi-exon genes (Zhang et al., 2017; Calixto et al., 2018; Jabre et al., 2019). IR represents the predominant AS event, often generating transcripts harboring premature stop codons, which are retained in the nucleus (Jia et al., 2020), degraded *via* nonsense-mediated mRNA decay (NMD) or translated into truncated proteins (Filichkin and Mockler, 2012; Syed et al., 2012; Chaudhary et al., 2019b). Some introns, named exitrons, present characteristics of protein-coding exons. Splicing of these exitrons modifies protein domains, affecting severely the protein function (Marquez et al., 2015). During different developmental stages and under environmental stress conditions, AS mechanisms contribute to modulate the ratio between functional and non-functional protein isoforms.

In tomato, Clark and colleagues (2019) integrated genomic and transcriptomic data to analyze the AS landscape, estimating that 65% of annotated protein-coding genes generate multiple transcript isoforms. Recent studies have characterized differential alternative splicing (DAS) in different tissues, in response to hormones or abiotic stresses, such as heat or drought (Zouine et al., 2014; Wang et al., 2016; Lee et al., 2020; Yang et al., 2020). Analysis of tomato seedlings, flowers and fruits showed that the number of splice variants per gene was higher in developing fruit compared to other organs, indicating distinctive, tissue-specific AS regulation (Sun and Xiao, 2015).

In addition, stress-responsive AS events may be differentially regulated depending on the tissue examined, as shown in maize (Thatcher et al., 2016). Indeed, Keller and colleagues observed extensive AS regulation in tomato pollen, a heat-sensitive tissue, in response to heat stress, where most of the transcript isoforms identified were partially or fully lacking functional domains (Keller et al., 2017).

Recently, AS pattern in response to heat and drought combination was studied in wheat as well as in tea plants, showing that combined stress can induce specific AS (Liu et al., 2018; Ding et al., 2020; Ding et al., 2022).

Here, we subjected ten tomato varieties to a combination of drought stress and low nitrate. By analyzing physiological and growth parameters, we selected T270 and T250 as sensitive and least affected by stress, respectively to analyze changes in gene expression and splicing landscape in two different tissues, root and leaf. We found key differences in terms of differentially expressed (DEGs) and spliced (DAS) genes, which thus highlighted the independent and specific role of AS in adaptation to stress condition.

## Materials and methods

### Plant material and growth conditions

Ten *Solanum lycopersicum* L. genotypes including reference genotype M82 (T162) and landraces from Southern Italy were used in this study. **Supplementary Datasheet S1** provides a list and main characteristics of the genotypes. Landraces under study were selected as tolerant in terms of yield to low water input conditions applied by local farmers, with the exception of T270, reported as sensitive. Seeds were germinated at a nursery (Coral Plant, Italy) in soil in a semi-controlled greenhouse, and irrigated daily with standard nutrient solution. At two true leaves stage, seedlings were transplanted to 15 L pots containing river sand, previously imbibed to field capacity with water, and maintained in semi-controlled conditions glasshouse. At transplant, plants were divided in eight blocks, each containing three to four replicates per genotype and watered for one week with control nutrient solution. One week after transplant, two blocks were used for each of four treatments: Control (10.2 mM $NO_{3}^{-}$ ; 100% water supply), Low Nitrate (2.88 mM $NO_{3}^{-}$ ; 100% water supply), Drought (50% water supply) and Combined Stress (2.88 mM $NO_{3}^{-}$ with 50% water supply). Plants belonging to Control and Low Nitrogen treatments were irrigated daily until pot saturation (100% water supply), while in Drought and Combined treatments the time of irrigation was halved to provide 50% water supply. Nitric acid (HNO_3_) was added to the nutrient solution to differentiate the treatments in terms of nitrogen supply. To maintain pH to 5.7± 0.1 in the different nutrient solutions, Na_2_CO_3_ was used to buffer excess acidity provided by HNO_3_ addition in control and drought stress conditions. Similarly, NaCl was added in low nutrient and combined stress solution to keep Na^+^ concentrations similar in all the treatments. Nutrient solution compositions were as follows: Control (Foliar drop 0.5 g/L, Brexil 0.05 g/L, KH_2_PO_4_ 0.1 g/L, Na_2_CO_3_ 0.675 g/L, NaCl 0.15 g/L, HNO_3_ (65%) 0.675 ml/L); Low Nitrate (Foliar drop 0.5 g/L, Brexil 0.05 g/L, KH_2_PO_4_ 0.1 g/L, NaCl 0.3 g/L, HNO_3_ (65%) 0.2 ml/L), Drought (Foliar drop 1 g/L, Brexil 0.1 g/L, KH_2_PO_4_ 0.2 g/L, Na_2_CO_3_ 1.35 g/L, HNO_3_ (65%) 1.35 ml/L) and Combined Stress (Foliar drop 1 g/L, Brexil 0.1 g/L, KH_2_PO_4_ 0.2 g/L, NaCl 0.6 g/L, HNO_3_ (65%) 0.2 ml/L)). A preventive phytosanitary treatment (PREVICUR^®^ Fungicide, Bayer CropScience) was conducted 9 days after transplant.

Plants were cultivated under stress conditions for 30 days and then harvested for molecular and biometric analyses. Leaf (youngest fully expanded leaf) and root samples for RNA extraction were collected and snap frozen in liquid nitrogen.

### Physiological parameter measurements

To monitor stress progression, chlorophyll content was measured on three leaves of each individual plant per treatment using Chlorophyll meter SPAD-502Plus (Konika Minolta, Japan). Stomatal conductance, monitored through porometer AP4-UM3 (Delta-T Devices, UK), and Leaf relative water content (LRWC) were measured on three replicates per genotype per treatment. For LRWC, excised leaves were immediately weighed obtaining the fresh weight (FW) and hydrated with distilled water for 24 hours to obtain the turgid weight (TW). Leaf samples were then oven-dried at 70°C for 72 hours and dry weight (DW) was measured. The LRWC percentage was calculated using the following equation: LRWC (%)=(FW-DW)/(TW-DW) ×100.

### Growth parameter measurements

At harvest, aerial part and roots were collected and separated. Roots were washed to remove residual sand. Plant height, shoot fresh weight, shoot dry weight, root fresh and dry weight, and leaf area were measured using 6 to 8 replicates per treatment per genotype. To obtain shoot dry weight, samples were oven-dried at 70°C until a stable weight was reached. Plant leaf area was measured on excised leaves of all plants using a scanning planimeter (LI – 3400 area meter, Licor).

### Statistical analysis

One way analysis of variance (ANOVA) within each genotype was carried out on physiological and growth parameters using the SPSS software package (SPSS 19 for Windows, SPSS Inc., an IBM Company, United States). When ANOVA indicated significant differences among treatments, mean separation was performed using the Duncan’s multiple range test. Different letters shown in figures indicate significant difference at *p*< 0.05.

### Heatmap construction

The cluster heatmap was produced with ClustVis tool (http://biit.cs.ut.ee/clustvis/) using Euclidean distance as the similarity measure and Ward as linkage rule (Metsalu and Vilo, 2015). Per each parameter and genotype, the percent variation induced by the different treatments compared to the control was used as data input.

### RNA sequencing

Total RNA was isolated from 100 mg of root and leaf samples using RNeasy Plant Mini Kit (Qiagen, Germany) according to manufacturer’s instructions.

For RNA deep sequencing, three biological replicates per genotype from control and combined stress treatments were used. The sequencing service was provided by Macrogen Europe (https://www.macrogen-europe.com/). Raw sequences are available at the National Center for Biotechnology Information Sequence Read Archive, bioproject PRJNA855575. The quantity and the quality of RNA (RNA integrity) were assured by using an Agilent Technologies (USA) 2100 Bioanalyzer. The poly-A tailed mRNA was collected with poly-T oligo beads of TruSeq stranded mRNA kit (Illumina, USA). The cDNA was synthesized from the randomly fragmented RNAs. The cDNA fragments were selected to obtain an optimal insert size of 200-400 bp. The sequencer specific adaptors and indexes were attached to the cDNA fragments to generate the libraries according to the TruSeq stranded mRNA kit instructions. The size and quantity of target-enriched libraries were confirmed by TapeStation D1000 Screen Tape (Agilent) and quantitative PCR following the standard Illumina qPCR Quantification Protocol. The normalized and pooled libraries were loaded into the flow cell of NovaSeq 6000 Illumina sequencer, where the bridged amplification reaction of these libraries occurred, and a series of images were captured from the extension of nucleotides possessing reversible fluorophore and termination properties.

### Transcriptomic and differential splicing analysis

Prior to further analysis, a quality check was performed on the raw sequencing data by using FastQC. (https://www.bioinformatics.babraham.ac.uk/projects/fastqc/), then low quality portions of the reads were removed with BBDuk (sourceforge.net/projects/bbmap/). The minimum length of the reads after trimming was set to 35 bp and the minimum base quality score to 25. On average, 51.8 million filtered reads were obtained per sample. The high quality reads were aligned against the *Solanum lycopersicum* reference genome sequence (ITAG4.0) with STAR aligner (version 2.5.0c, Dobin et al., 2013). On average 86% of the reads could be uniquely mapped to the reference genome. FeatureCounts (version 1.4.6-p5, Liao et al., 2014) was used together with the ITAG4.0 annotation to calculate gene expression values as raw read counts. Normalized TMM and FPKM values were calculated. All the statistical analyses were performed with *R* with the packages HTSFilter (Rau et al., 2013) and edgeR (Robinson et al., 2010). The first step was the removal of not expressed genes and the ones showing too much variability. The HTSFilter package was chosen for this scope, which implements a filtering procedure for replicated transcriptome sequencing data based on a Jaccard similarity index. The “Trimmed Means of M-values” (TMM) normalization strategy was used. The filter was applied to the different experimental conditions in order to identify and remove genes that appear to generate an uninformative signal. The overall quality of the experiment was evaluated, on the basis of the similarity between replicates, by a Principal Component Analysis (PCA) using the normalized gene expression values as input. The differential expression analysis was performed to identify the genes that are differentially expressed in all comparisons. Only genes with an FDR equal or lower than 0.05 were considered as Differentially Expressed Genes (DEGs).

In order to identify the number of different splicing events the software rMATS (V 3.2.5, Shen et al., 2014) was used. Prior to further analysis, the high quality reads were aligned against the reference genome with STAR aligner (version 2.5.0c), with Local Mapping and in double pass. rMATS was then used with the following options: -t “paired” –libType “fr-firststrand” –readLength 150 –variable-read-length –anchorLength 15 –allow-clipping –novelSS –mil 3 –mel 13575. The –novelSS option was used since the ITAG4.0 annotation does not report splicing variants. An FDR filter of<=0.05 and an absolute minimum Inclusion Difference of 0.25 was used to detect significant differences in splicing. Furthermore, for further analysis were considered events supported by at least 20 reads as the sum of reads in all treatments in each of the pairwise comparisons for each of the including form and the skipping form. For the DEGs and significantly different splicing events, a Gene Ontology Enrichment Analysis (GOEA) was performed to identify the most enriched Gene Ontology (GO) categories across the down- and up-regulated genes following the method described in Tian et al. (2017). The Gene Ontology annotation was updated using the software Pannzer2 (Toronen et al., 2018) providing the FASTA file of the proteins as input and selection the following options: Minimum query coverage 0.4 or minimum subject coverage 0.4 and minimum alignment length 40. Transcriptomic and differential splicing analysis was performed by Sequentia Biotech (http://www.sequentiabiotech.com). Differential splicing results were validated through RT-qPCR. Primers used are listed in **Supplementary Datasheet S2**.

## Results

### Physiological response to single and combined stress

**Figure 1:**
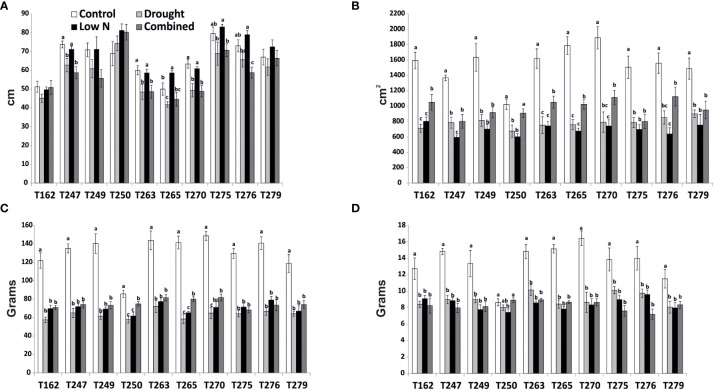
Biometric parameters measured at the end of the experiment in the four treatments in all genotypes. **(A)** Height; **(B)** Leaf area; **(C)** Shoot fresh weight; **(D)** Shoot dry weight. Values indicate mean ± SE (n≥6). Different letters indicate significant difference within each genotype at *p*< 0.05 (Duncan test).

**Figure 2:**
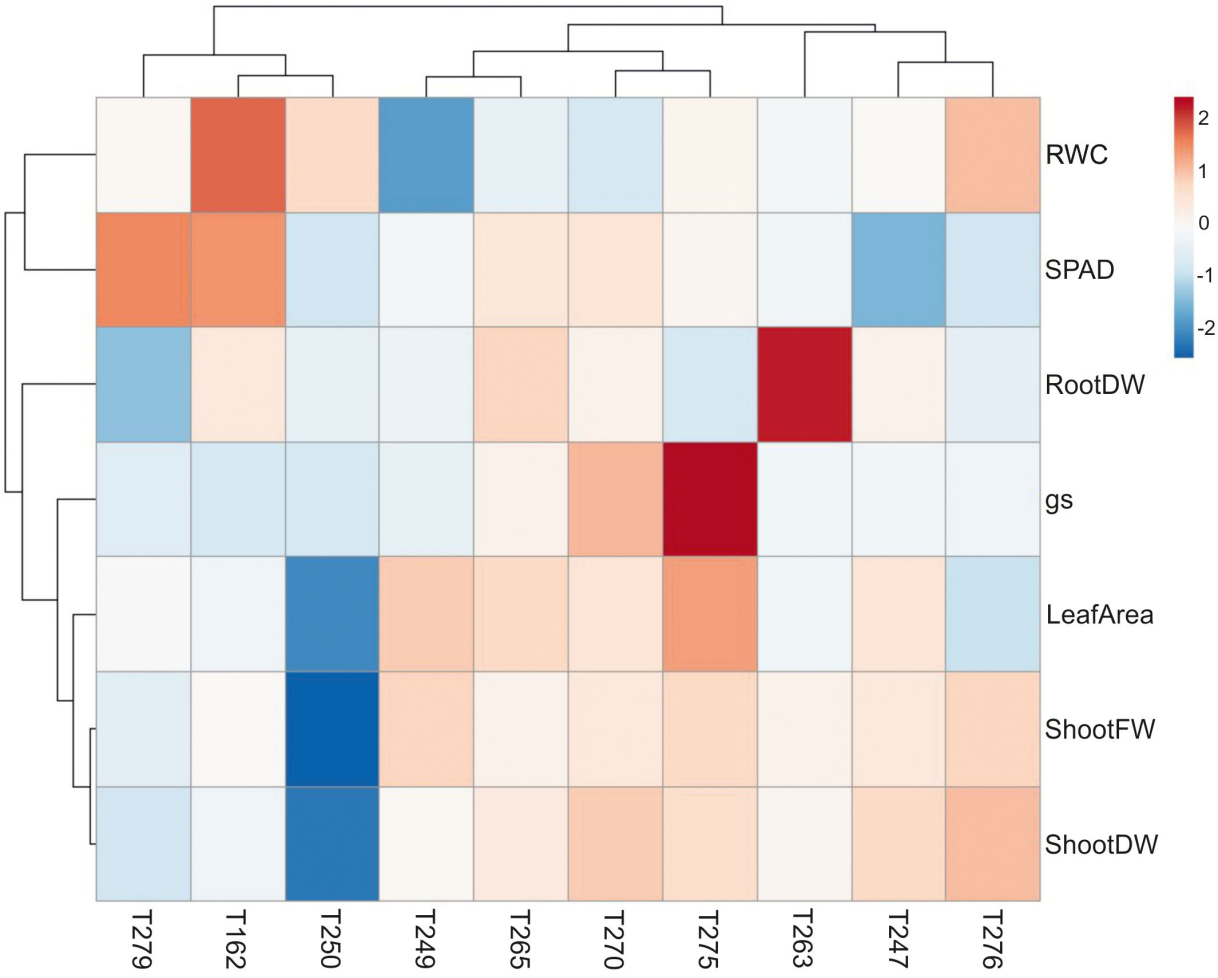
Heatmap constructed through ClustVis (http://biit.cs.ut.ee/clustvis/) of percentage differences in physiological and biometric parameters measured in plants of the 10 genotypes analysed (See **Supplementary Datasheet S1**) subjected to combined stress versus control condition. The red-blue color gradient scaling is indicated, where red and blue colors indicate highest and lowest variations in values measured in stressed plants compared to controls, respectively. Rows and columns are clustered through correlation distance and average linkage. RootDW, Root Dry Weight; gs, Stomatal Conductance; ShootFW, Shoot Fresh Weight; ShootDW, Shoot Dry Weight; RWC, Leaf Relative Water Content; SPAD, Leaf SPAD Values.

**Figure 3:**
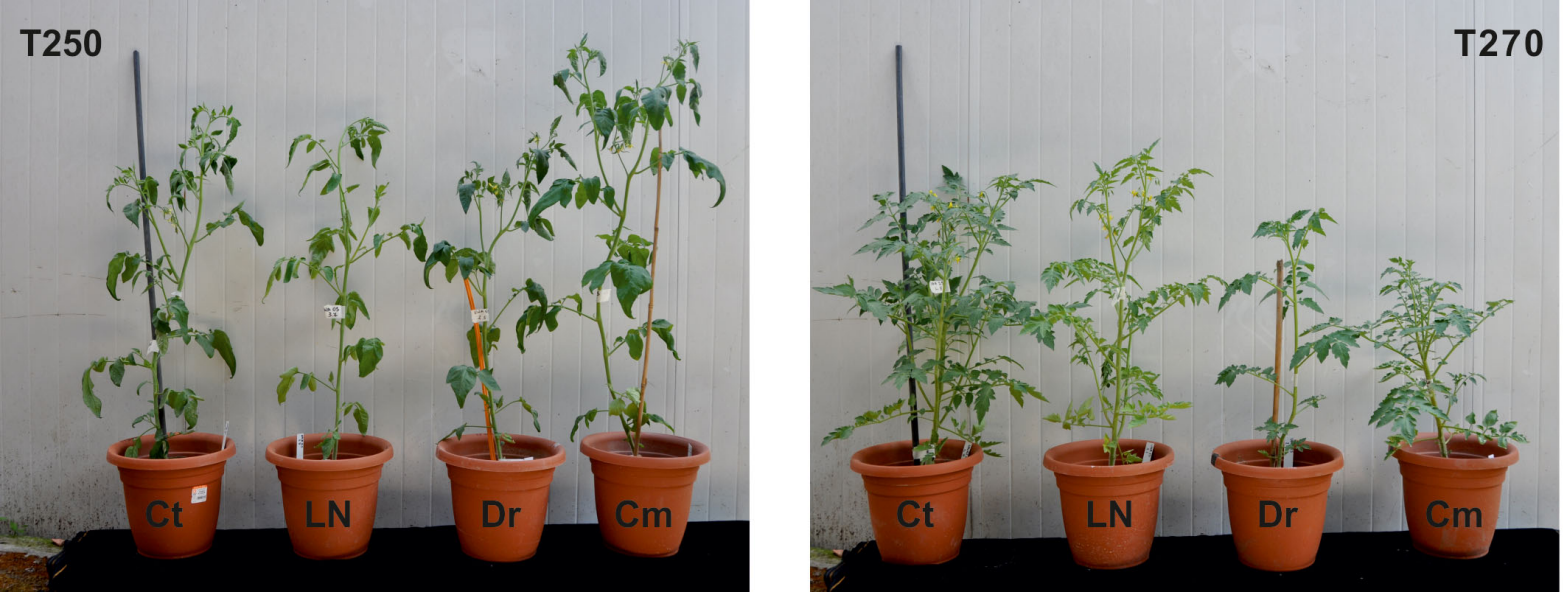
Representative plants of genotypes T250 (left) and T270 (right) grown for 24 days in Control (Ct), Low Nutrient (LN), Drought (Dr) or Combined stress (Cm) condition.

To identify tomato genotypes with contrasting responses to combined stress, we cultivated 10 genotypes, including landraces and varieties (**Supplementary Datasheet S1**) in single pots in four different conditions: Control, Low Nitrate, Drought and Combined Stress. Physiological measurements confirmed a lower chlorophyll content in low nitrate and combined stress treatments, and a low stomatal conductance in plants subjected to drought or combined stress (**Supplementary Figure S1**). After 30 days of growth under stress, biometric parameters were measured (**Figure 1**). Concerning plant height, genotypes T162, T249, T250 displayed no difference between the treatments (**Figure 1A**). The remaining six genotypes showed significantly lower height in drought and combined stress treatment compared to control; genotype T270 showed the biggest difference in height between combined (49 cm) and control treatment (63 cm) (**Figure 1A**). Leaf area (LA) measured in stressed plants was lower than that of the control treatment, in all genotypes; however, single stresses resulted in a bigger penalty in the leaf area than combined stress (**Figure 1B**). Notably, T250 was the only genotype with virtually no difference in LA in combined stress (909 cm^2^), compared to control (1024 cm^2^), whereas T275 was the most affected, with a LA in combined stress approximately half that of the control (**Figure 1B**). Shoot fresh and dry weight of all genotypes were reduced in the stress treatments (**Figures 1C, D**); T250 was least affected by stress (-12.68% shoot fresh weight and +3% shoot dry weight in combined stress) (**Figures 1C, D**). On the opposite end, T276 and T270 showed the biggest reduction in weight in combined stress compared to control treatment (**Figure 1D**). Thus, combined stress had a different impact on growth parameters depending on the genotype tested. As summarized in the heatmap provided in **Figure 2**, extensive variations in percentage differences of physiological and biometric parameters measured in combined stress *vs.* control treatment were observed, with T250 showing little or no reduction in several biometric parameters, and was thus considered tolerant, whereas T270, like other genotypes, was severely affected by combined stress and was thus selected as sensitive genotype. These two genotypes showed obvious differences in root systems, with T250 having a smaller root and T270 a more developed root system (**Table 1**). In addition, T250 had a lower root/shoot ratio, stable in control and combined stress conditions, whereas T270, similarly to T276 and T263, was characterized by a higher root/shoot ratio showing a significant increase in combined stress (**Table 2**). Thus, T250 and T270 (**Figure 3**) were selected for further molecular analyses.

**Table 1:** Root biometric parameters in control treatment.

Genotype	Root fresh weight (g)	Root dry weight (g)
**T250**	17.32 ± 1.37	1.47 ± 0.13
**T270**	64.87 ± 2.59	4.87 ± 0.32

Values indicate mean ± SE (n≥6).

**Table 2:** Root/shoot ratio in control and combined stress treatments.

Genotype	Control	Combined stress
**T162**	0.29 ± 0.04 (b)	0.48 ± 0.08 (a)
**T247**	0.30 ± 0.03 (b)	0.58 ± 0.07 (a)
**T249**	0.16 ± 0.02 (b)	0.30 ± 0.04 (a)
**T250**	0.17 ± 0.01 (n.s.)	0.20 ± 0.02 (n.s.)
**T263**	0.34 ± 0.03 (n.s.)	0.38 ± 0.05 (n.s.)
**T265**	0.24 ± 0.02 (n.s.)	0.38 ± 0.07 (n.s.)
**T270**	0.30 ± 0.03 (b)	0.57 ± 0.02 (a)
**T275**	0.20 ± 0.02 (b)	0.46 ± 0.07 (a)
**T276**	0.30 ± 0.03 (b)	0.71 ± 0.08 (a)
**T279**	0.20 ± 0.03 (b)	0.47 ± 0.06 (a)

Values indicate mean ± SE (n≥6); different letters indicate significant difference within each genotype at p< 0.05 (Duncan test). N.s., non significant.

### Impact of combined stress on root and leaf gene expression

To investigate gene expression changes elicited by combined drought stress and low N in tomato, and responsible for the phenotypic and physiological changes observed, a transcriptomic analysis was performed on T250 and T270, with the aim of identifying common or specific responses. Given the importance of the root systems in the perception of water/nutrient availability and their uptake, RNA was extracted and sequenced from roots in addition to leaves, collected 30 days after stress initiation. Out of 34,075 genes annotated in the reference genome sequence (ITAG4.0), ~ 22, 000 unigenes on average were expressed (FPKM > 1), of which ~ 52% were found in leaves and ~ 57% in roots of both genotypes (**Supplementary Figure S2A**). Venn diagrams were then constructed, highlighting that over 90% of expressed genes in control conditions were common to the two genotypes in both tissues, whereas ~ 5% on average were specific (**Supplementary Figure S2B**). Despite the high number of shared genes, gene expression levels were different in the two genotypes, especially in leaves. Indeed, by performing the differential expression analysis in the pairwise comparison T250 *vs.* T270, 1,086 differentially expressed genes (DEGs) were found in leaves (588 up-regulated and 498 down-regulated), whereas only eight genes (six up-regulated and two down-regulated) were annotated as DEGs in roots, suggesting that the root transcriptomes in the two genotypes were comparable (**Supplementary Datasheet S3**). Gene Ontology Enrichment Analysis (GOEA) revealed several GO terms involved in stress response-related categories as significantly enriched (FDR< 0.05) in the tolerant genotype, along with categories related to plant development (**Supplementary Figure S2C**).

**Figure 4:**
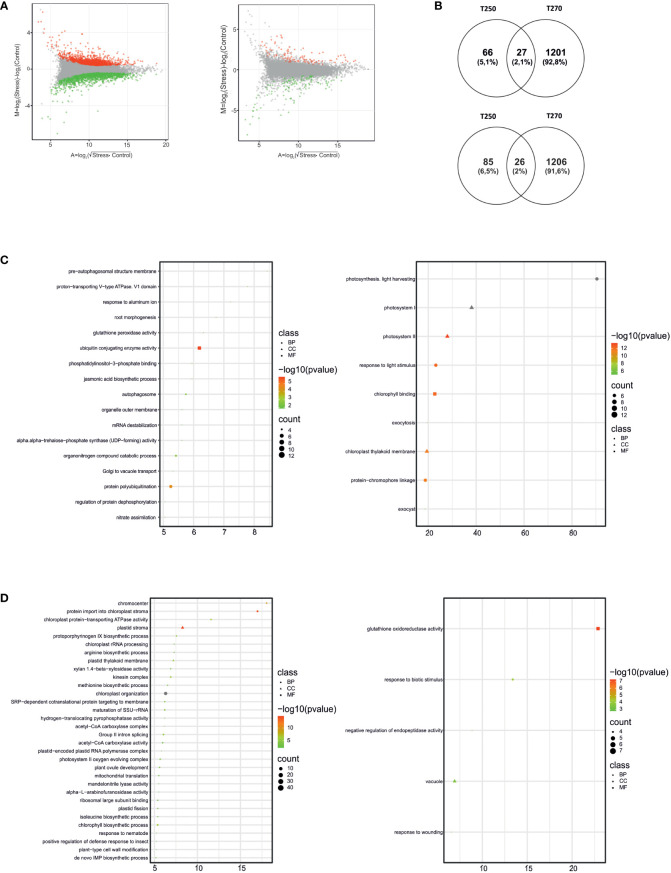
Differentially expressed genes (DEGs) and enriched Gene Ontology terms in leaves of T250 and T270 subjected to combined stress. **(A)** MA plots depicting the distribution of DEGs (green dots, down-regulated, red dots, up-regulated) in T270 (left) and T250 (right) leaves. X-axis: log_2_ mean expression across treatments; Y-axis: log_2_ expression fold change in combined stress *vs.* control treatments. **(B)** Venn diagrams depicting number and overlap of up-regulated (upper panel) and down-regulated (lower panel) DEGs in leaves of combined stress-treated T250 and T270. The diagrams were drawn using the online tool Venny (Oliveros, 2007-2015); **(C, D)** Plots showing enriched GO terms in leaf DEGs up **(C)** or down-regulated **(D)** in T270 (left) or T250 (right). Symbols indicate GO categories: MF, Molecular Function; CC, cellular compartment, BP, biological process. Symbol sizes are proportional to the gene count, whereas colors represent FDR values< 0.05. X-axis: enrichment score. Categories with ES> 5 and gene count > 4 are shown.

Pairwise comparisons were also carried out in stressed samples *versus* their respective control (**Supplementary Datasheet S4**). As shown in **Figure 4A**, responses in terms of the total number of DEGs elicited by combined stress in leaves were different in the two genotypes. Indeed, 2,460 DEGs were identified in the leaves of the former genotype, whereas only 204 were found in the latter (**Supplementary Datasheet S4**). GOEA and Venn diagrams highlighted how gene expression changes elicited in leaves were genotype-specific, with less than 30 genes being up- or down-regulated and common between T250 and T270 (**Figures 4B-D**). Among enriched GO terms, different categories related to photosynthesis were differentially regulated in the two genotypes. In T250, GO terms such as “photosynthesis”, light harvesting” and “response to light stimulus”, “photosystem I”, “photosystem II”, “chlorophyll binding” were enriched among up-regulated DEGs, including several genes encoding Chlorophyll a-b binding proteins. By contrast, in T270 leaves, several GOs related to catabolic processes were enriched among up-regulated genes, including autophagy and protein-ubiquitination related GOs as well as an “mRNA destabilization” (**Figure 4C**). GO “chlorophyll biosynthetic process” was enriched among down-regulated genes together with several categories related to plastid function and biology (**Figure 4D**).

Among the other genotype-specific changes in the sensitive T270, 30 genes coding for histones (histone H1, histone H2A, histone H2B, histone 3 and histone 4) were down-regulated, in addition to ten genes encoding heat shock proteins and one *Pyrroline-5-carboxylate reductase*; this latter known to be involved in proline biosynthesis (**Supplementary Datasheet S4**). Exclusively up-regulated genes in T270 leaf included 12 nitrate transporters, five heat shock proteins, three heat shock factors, three PYLs and two LEAs, whereas only 66 genes were exclusively up-regulated in T250, including a *Nitrate Transporter* in addition to the Chlorophyll a/b binding proteins described above (**Supplementary Datasheet S4**).

**Figure 5:**
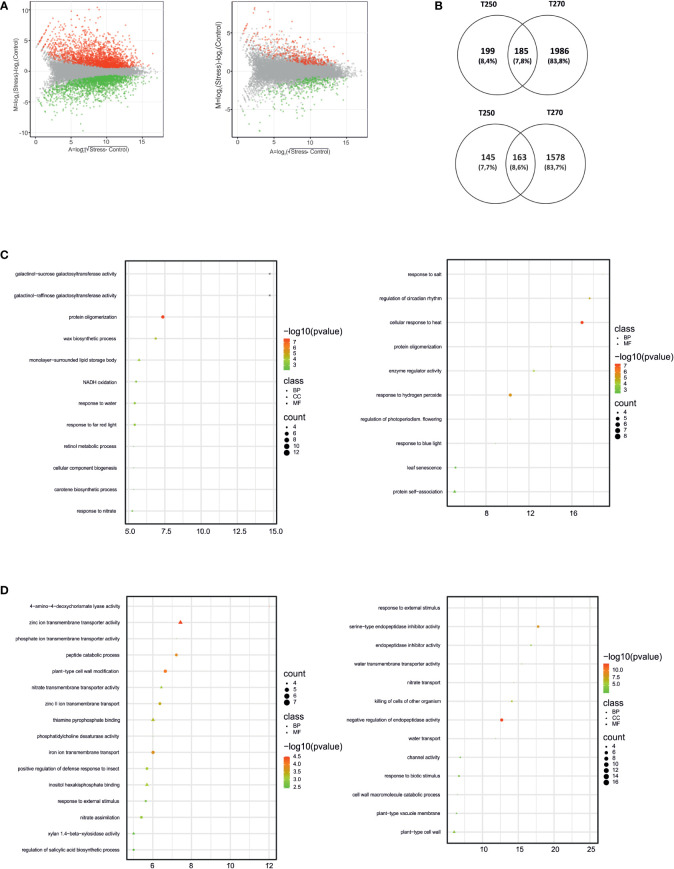
Differentially expressed genes (DEGs) and enriched Gene Ontology terms in roots of T250 and T270 subjected to combined stress. **(A)** MA plots depicting the distribution of DEGs (green dots, down-regulated, red dots, up-regulated) in T270 (left) and T250 (right) roots. X-axis: log_2_ mean expression across treatments; Y-axis: log_2_ expression fold change in combined stress *vs.* control treatments. **(B)** Venn diagrams depicting number and overlap of up-regulated (upper panel) and down-regulated (lower panel) DEGs in roots of combined stress-treated T250 and T270. The diagrams were drawn using the online tool Venny (Oliveros, 2007-2015); **(C, D)** Plots showing enriched GO terms in root DEGs up **(C)** or down-regulated **(D)** in T270 (left) or T250 (right). Symbols indicate GO categories: MF, Molecular Function; CC, cellular compartment, BP, biological process. Symbol sizes are proportional to the gene count, whereas colors represent FDR values< 0.05. X-axis: enrichment score. Categories with ES> 5 and gene count > 4 are shown.

As was true for leaves, responses in terms of the total number of DEGs in roots was higher in the susceptible genotype T270 compared to the tolerant T250, since 3,912 and 692 DEGs were identified, respectively (**Supplementary Datasheet S4**; **Figure 5A**). GOEA analysis and Venn diagrams revealed interesting genotype-specific and shared features (**Figures 5B-D**). Among genotype-specific changes, GOs related to galactinol galactosyltransferase activity were up-regulated in susceptible T270, along with “response to nitrate” and “response to water”, whereas “regulation of circadian rhythm” and “response to heat” were induced in the tolerant T250 (**Figure 5C**). Concerning GOs enriched among down-regulated genes, “water transport” and “nitrate transport” were identified in T250, whereas “nitrate assimilation” was peculiar to T270 (**Figure 5D**), pointing to different responses of the root systems in the two genotypes. The sensitive genotype showed 1,986 exclusively induced DEGs (**Figure 5B**), including osmotic stress responsive genes such as *HSPs*, *LEAs*, *ERDs*, *AREB* Transcription factors, and a *Delta-1-pyrroline-5-carboxylate synthase* (*P5CS*) that catalyzes the first two steps in proline biosynthesis. Different candidates belonging to the same gene families were also identified in T250 roots but in lower number (**Supplementary Datasheet S4**). In addition, as shown in **Figure 5B**, root tissues shared 163 and 185 commonly down and up-regulated genes, respectively. Among the former dataset, a gene coding for a putative Wuschel protein was in common between T250 and T270, together with three nitrate transporters and ABA receptor *PYL4*. By contrast, among the second dataset, six HSPs, six HSFs, three NRTs, two LEAs, one dehydrin, and ABA receptor *PYL10* were found (**Supplementary Datasheet S4**).

### Alternative splicing regulation

**Figure 6:**
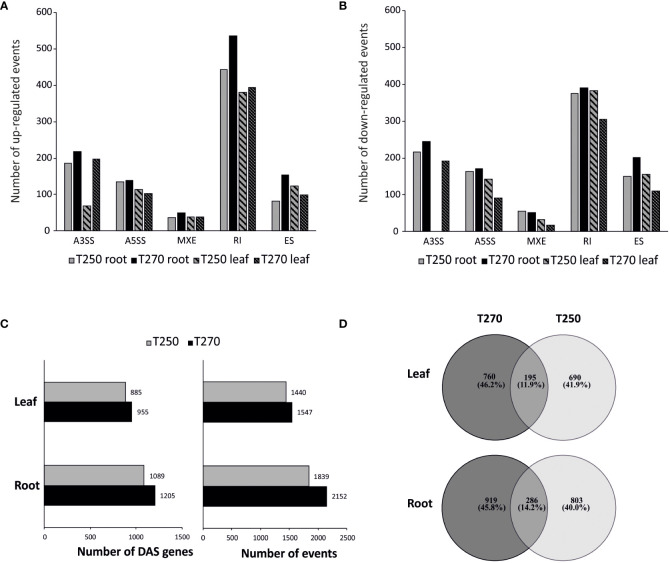
Alternative splicing (AS) regulation in root and leaf of T250 and T270 under combined stress condition. **(A, B)** Bar graph of the number of differential up- **(A)** and down-regulated **(B)** AS events. **(C)** Number of genes concerned by DAS (left) and DAS events (right). **(D)** Venn diagram depicting the number of genotype-specific and common (DAS) genes.

We investigated the common and genotype-specific alternative splicing (AS) regulation in response to combined stress in T250 and T270 leaf and root tissue. Multivariate Analysis of Transcript Splicing software (rMATS) was used to investigate AS events, including intron retention (IR), alternative 3’ or 5’ splice site (A3SS; A5SS), exon skipping (ES) and mutually exclusive exon (MXE) (**Supplementary Datasheet S5**). Pairwise comparisons identified differential alternative splicing (DAS) events, and the concerned genes, in both control and stress conditions (**Figure 6**, **Supplementary Datasheets S6-S11**). The DAS events identified through RNA-seq were validated by RT-qPCR, and the presence of non-canonical splice variants was verified in additional genotypes (**Supplementary Figure S3**).

In all pairwise comparisons, IR was the most abundant category, consistent with previous studies in plants (Filichkin et al., 2010; Chaudhary et al., 2019a), whereas MXE was the least represented (**Figures 6A B**; **Supplementary Figure S4A**).

When genotypes were analyzed under control conditions, 934 and 1,210 events were identified in root and leaves, respectively, and annotated as DAS in the tolerant genotype compared to the susceptible one (**Supplementary Datasheets S6-S7**). GOEA analysis on this subset revealed GO terms involved in the regulation of gene expression, RNA binding, nucleoplasm and peroxisome as significantly enriched in both leaves and roots of the tolerant genotype T250 compared to the susceptible T270 (**Supplementary Figures S4B, C**).

In stress conditions, we observed a substantial number of genes subjected to DAS under stress in both genotypes. Consistent with DEGs, a higher number of DAS events were detected in roots (**Figure 6C**). In particular, we detected 2,152 and 1,839 DAS events in T270 and T250 roots, respectively, compared to 1,547 and 1,440 in leaves. Less than 30% of genes concerned by DAS were shared by the two genotypes in both tissues (**Figure 6D**). In detail, the percentage of events common to the two genotypes was lower than 8% for A3SS and A5SS, and lower than 10% in the case of SE, IR and MXE. For all categories, the common events were lower in leaves compared to roots (**Table 3**). The fraction of genotype-specific A3SS events was highest in T270 leaves (84%) compared to T250 leaves (14%), whereas similar percentages were found in roots (50% in T270 and 44% in T250) (**Table 3**).

**Table 3:** Number of shared and genotype-specific transcripts subjected to each of the five splicing types of event in T250 and T270 leaf and root.

	Splicing event	T250 total	T270 total	T250 specific	T270 specific	Common
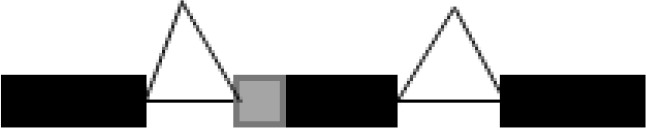	**A3SS leaf**	57	307	49	299	8
				14%	84%	2%
	**A3SS root**	315	352	272	309	43
				44%	50%	7%
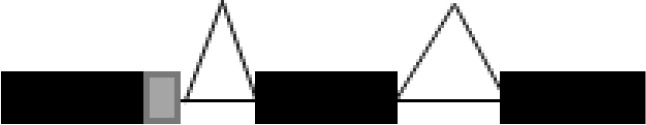	**A5SS leaf**	195	161	177	143	18
				52%	42%	5%
	**A5SS root**	230	234	194	198	36
				45%	46%	8%
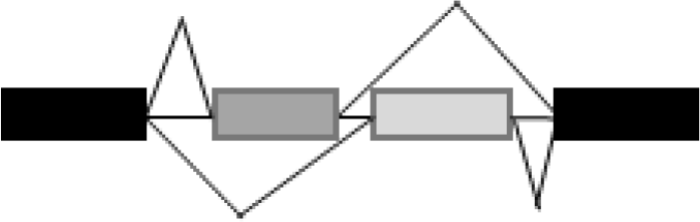	**MXE leaf**	44	25	38	19	6
				60%	30%	10%
	**MXE root**	50	50	41	41	9
				45%	45%	10%
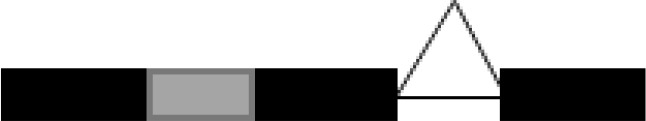	**IR leaf**	688	630	570	512	118
				48%	43%	10%
	**IR root**	741	814	599	672	142
				42%	48%	10%
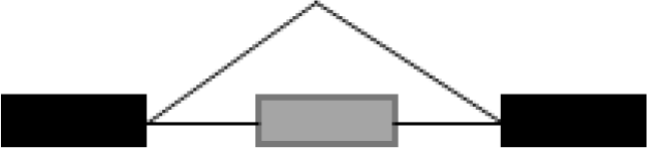	**ES leaf**	151	131	128	108	23
				49%	42%	9%
	**ES root**	138	174	109	145	29
				39%	51%	10%

A3SS, alternative 3’ splice site; A5SS, alternative 5’ splice site; MXE, mutually exclusive exon; IR, intron retention; ES, exon skipping.

To better understand the correlation between gene expression and RNA processing regulation, we compared DAS genes to DEGs. Little overlap was detected between DAS and DE genes in both genotypes. In T250 in particular, only 3 and 34 differentially regulated genes in leaf and root respectively, were also regulated by DAS (**Table 4**; **Supplementary Datasheet S12**).

**Table 4:** Number and fraction of DEGs and DAS genes in T250 and T270 leaf and root in combined stress condition.

Genotype	Tissue	Number of DEGs	Number of DAS	DEGs Specific	DAS Specific	Common
**T250**	leaf	204	885	201	882	3
				(18.5%)	(81.2%)	(0.3%)
	root	692	1089	658	1055	34
				(37.7%)	(60.4%)	(1.9%)
**T270**	leaf	2460	955	2348	843	112
				(71.1%)	(25.5%)	(3.4%)
	root	3912	1205	3721	1014	191
				(75.5%)	(20.6%)	(3.9%)

GOEA analysis was performed to investigate the biological functions and the cellular component of genes that undergo DAS in response to combined drought and low nitrogen stress (**Supplementary Figures S4-S5**). In leaves, categories such as “response to abiotic stimulus” and “photosynthesis” were enriched among DAS genes in T270, whereas “cellular homeostasis” and “cell growth” terms were enriched in T250 (**Supplementary Figure S5**). In roots, categories such as “peroxisome”, and “response to extracellular stimulus” were specific to the tolerant T250, whereas “endomembrane system” and “tropism” were found only in T270.

Interestingly, we identified many genes involved in *TOR* pathway, major regulatory pathway controlling plant growth. For example, a transcript retaining intron of putative tomato *TOR* orthologous gene (*Solyc01g106770.4*) was down-regulated in T250 roots. Additional orthologous genes involved in *TOR* pathway such as *LST8* and *RAPTOR2* were also differentially spliced in T250.

Among other genes that underwent DAS under combined stress, we detected alteration in key players involved in phytohormone metabolism, transport and signaling, abscisic acid, ethylene and auxin-related pathways. For example, numerous protein phosphatases belonging to the *PP2C* family, such as *SlPP2C30 (Solyc03g121880)*, a member of group A of PP2C (Qiu et al., 2022), underwent alternative splicing regulation in T250 or T270 roots. This is noteworthy since clade A *PP2Cs* are negative regulators of ABA responses and are involved in several physiological and biochemical processes during biotic and abiotic stress responses (Lee and Luan, 2012).

Several splice variants of stress and hormone-related transcription factors were also detected, including *MYBs*, *bHLHs*, *bZIPs*, *WRKYs*, *Heat Shock Transcription factors* and *Auxin Response Factors*, indicating that AS regulates their activity in response to combined stress.

Similarly, numerous intron-retaining variants of splicing factors, such as Serine/Arginine-rich-splicing factor *SR46a* (*Solyc06g076670*), *RS40* (*RS41*, *Solyc11g072340*), *SCL30A* (*SCL29*, *Solyc11g072340*) were differentially spliced.

## Discussion

Drought stress and low nitrate availability limit tomato plant growth, as shown here and in previous reports (*e.g.* Iovieno et al., 2016; Abenavoli et al., 2016). Because reduction of inputs in agriculture and improvement of plant stress tolerance are becoming increasingly urgent issues, we have focused on the combined drought/low N stress condition, analyzing tomato responses in terms of morpho-physiological and transcriptomic changes in different genotypes. The combined stress condition applied had a different impact depending on the biometric or physiological parameter and the genotype examined, indicating that strategies to withstand simultaneous stresses may vary among genotypes (**Figures 1**-**3**, **Tables 1**-**2**). Combined stress had little impact on leaf area and shoot weight of T250, whereas growth of T270 was severely hindered. Root systems of the two genotypes were dramatically different, with T250 having a small root apparatus in terms of dry weight, and a lower root/shoot ratio compared to T270 or other genotypes analyzed here (**Table 2**). An increase in root/shoot ratio is a common response to limited nitrate and water availability, and is a trait often observed in tolerant genotypes (Koevoets et al., 2016; Moles et al., 2018). T270 increased the root/shoot ratio in response to combined stress, whereas this parameter remained low and stable across the treatments in the tolerant T250, thus suggesting that T250 may possess a root system that maybe pre-adapted to stressful environments that does not require adaptive modifications. In addition, T250 shows the lowest biomass accumulation also in control conditions, which may aid in maintenance of tissue water and N status under stressful conditions (Araus et al., 2020).

The analysis of transcriptomic changes of T250 and T270 leaf and root in control and under combined drought stress and nitrogen deficiency conditions gave a comprehensive view of differential expression and alternative splicing of transcripts (**Figures 4**-**6**, **Supplementary Datasheets S3-S12**). In both genotypes, combined stress caused most transcriptomic changes in roots (**Figures 4**-**5**), consistent with a previous work carried out in spinach (Joshi et al., 2020).

Cytokinins (CKs) contribute to modulation of lateral root proliferation and mediate one of the major long-distance root to shoot nitrogen signaling pathways (Ruffel et al., 2011; Zhang et al., 2020), starting from adenosine phosphate-isopentenyl-transferase (IPT), which catalyzes the initial and rate limiting step of CKs biosynthesis. In our RNAseq results (**Supplementary Datasheet S4**), a gene coding for IPT (*Solyc04g007240.1*) was present and up-regulated only in T270 root, while another *Solyc09g064910.1* was repressed in T270 leaf, possibly indicating enhanced CK synthesis in T270 root. In addition, in T270, eight genes implicated in the CKs activation pathway were differentially expressed, indicating a CK-dependent modulation of transcription, which could possibly contribute together with other pathways to the increase in root/shoot ratio observed in T270. CKs are implicated in the maintenance of the stem cell homeostasis in the shoot apical meristem, by inducing *WUSCHEL* (*WUS*) (Chickarmane et al., 2012), and repressing *CLAVATA3* (*CLV3*) expression (Nimchuk et al., 2015). *WUS* acts as a positive regulator for the expression of *CLV3*, which in turn negatively regulates the meristem size by suppressing *WUS* expression (Ikeda and Ohme-Takagi, 2014). *CLV3* (Solyc11g066120.3) and *WUS* (Solyc11g072770.2) were both down-regulated in T270 root, consistent with the markedly reduced growth of T270 in combined stress condition compared to T250.

ABA-dependent pathways of stress response are implicated in both drought and nitrogen deficiency (Machado et al., 2022). Interestingly, 10 genes encoding PYR/PYL ABA receptors were differentially expressed in T270 root, most of them down-regulated, compared to only 2 in T250, suggesting that repression of expression of PYLs may contribute to stress signal desensitization (Ali et al., 2021) in T270.

Drought stress and nitrogen deficiency also impair photosynthesis (Iovieno et al., 2016; Mu and Chen, 2021); under drought in particular, several GO categories related to photosynthesis were enriched in clusters showing down-regulation in drought stressed samples, including those containing light-harvesting chlorophyll a/b-binding protein coding genes (*LHCBs*, Iovieno et al., 2016). In T250 exposed to combined stress, “Photosynthesis, light harvesting” and “Response to light stimulus” GOs, as well as 12 *LHCBs* were up-regulated in leaf, indicating an active photosynthetic machinery. It is reported that the Arabidopsis *LHCBs* are positively involved in guard cell signaling in response to ABA, and they may affect ABA signaling partly by modulating ROS homeostasis (Xu et al., 2012). In addition, over-expression of several *LHCBs* was shown to improve stress tolerance (Zhao et al., 2020). The observed up-regulation of *LHCBs* in T250 leaves may thus contribute to explain the tolerance traits displayed by this genotype.

In T270 leaves autophagy and protein-ubiquitination related terms and mRNA destabilization terms were enriched among up-regulated genes, which may indicate a degradation of cytoplasmic, protein and RNA material to aid survival under stress (Su et al., 2020).

Down-regulation of histone coding genes was previously observed in tomato plants exposed to drought stress and was correlated to a reduction of cell division and growth (Iovieno et al., 2016). In our RNAseq results, down-regulation of 36 histone *loci* was observed in susceptible T270, indicating that this may contribute to the observed growth arrest under stress.

Alternative splicing (AS) is among the main co- and post-transcriptional mechanisms involved in plant adaptation to suboptimal environmental conditions (Laloum et al., 2018; Punzo et al., 2020). Here, we have identified a high number of combined stress-related splice events in root and leaf of genotypes T250 and T270. Interestingly, compared to the low number of DEGs, the alternative splicing profile of T250 changed strikingly under combined stress (**Table 4**), suggesting that AS might represent the prevalent mechanism of transcription regulation in this genotype.

In tomato, the key regulator of heat stress response *HsfA2* (Fragkostefanakis et al., 2016) is also induced in response to water deprivation, with higher up-regulation in root compared to leaf (Mishra et al., 2021). Consistent with this report, we observed severe up-regulation of *HsfA2* in roots of both genotypes. In addition, *HsfA2* was recently shown to be alternatively spliced in response to heat stress, with isoform abundance being temperature-dependent (Hu et al., 2020; Broft et al., 2022). The two main variants, *HsfA2-II* and *HsfA2-Iα*, characterized by splicing or retention of intron 2 respectively, were detected with different abundance in wild or modern tomato accessions. *HsfA2-I* was mainly observed in wild tomatoes and proposed to increase their capacity to rapidly acclimate against severe heat stress compared with modern cultivars (Hu et al., 2020). Interestingly, we observed down-regulation of *HsfA2-Iα* in T250 roots, suggesting rapid acclimation to stress of this genotype.

It has been shown that tomato HSFs regulate the expression of several Serine/arginine-rich (SR) proteins, which are important regulators of alternative splicing (Rosenkranz et al., 2021). SR genes are subject to alternative splicing themselves. In Arabidopsis, abiotic stresses, such as high temperature and salinity as well as hormones, severely affect splicing of SR transcripts (Palusa et al., 2007; Duque, 2011). In tomato, 17 SR protein-coding genes were identified. Most of them showed a reduction in protein levels under heat stress (Rosenkranz et al., 2021). We observed DAS of numerous members of SR family under combined stress, including *Solyc06g076670* (*Sl-SR46a*), orthologue of Arabidopsis *SR45a*.

SR45a acts in Arabidopsis at the early stage of spliceosome assembly through the interaction with U1-70 K and U2AF35b (Tanabe et al., 2009; Day et al., 2012), and is involved in different environmental stress response, such as high-light irradiation and salinity (Tanabe et al., 2007; Li et al., 2021). Two splice variants of *SR45a* are induced by salt stress, *SR45a-1a* encoding full-length functional protein and *SR45a-1b* encoding a truncated isoform lacking the C-terminal RS domain. SR45a-1a interacts with the cap-binding protein 20 (CBP20) which regulates the expression and splicing of salt-related genes. Interestingly, the truncated isoform SR45a-1b promotes the interaction between SR45a-1a and CBP20, increasing the plant salt-stress tolerance (Li et al., 2021).

The tomato *Sl-SR46a* is highly expressed in response to heat stress due to the presence of HS elements (HSEs) in the promoter, that are bound by HSFs (Rosenkranz et al., 2021). Interestingly, we detected down-regulation of the ES event of *SR46a* in T250 leaf compared to T270 in control condition. This predicted exon is located in the intron 5 region and contains a predicted premature terminator codon when included in the transcript. Thus, we speculate that the identified DAS event in *Sl-SR46a* may influence the T250 stress tolerance through a regulatory mechanism similar to Arabidopsis.

Auxin is mainly involved in the regulation of plant growth and development, however their roles in abiotic stress response were reported in different species (Jain and Khurana, 2009; Ha et al., 2013; Sekhwal et al., 2015). In tomato, several Auxin Response Factors (ARF) are differentially expressed under drought, salt or flooding condition both in leaves and roots (Bouzroud et al., 2018). We mainly observed differential regulation of several ARFs in T270 root. Interestingly, these factors are also regulated by AS. Studies on tomato fruit set revealed that over 30% of ARF genes undergo AS, and all the analyzed splice variants generated frame shift that creates a premature stop codon (Zouine et al., 2014). We detected several ARF splicing variants mainly in T250, such as *ARF4*, *ARF8A*, *ARF9B*, indicating that AS regulates these factors also in response to combined stress. In Arabidopsis, several ARF splice variants were characterized. The two isoforms of the *ARF4*, *ARF4* and *ΔARF4*, lacking sequence of exon, showed different function during carpel development (Finet et al., 2013). A splicing variant of *ARF8.2*, *ARF8.4*, characterized of retention of intron 8, regulates stamen elongation and endothecium lignification by directly activation of AUX/IAA19 and MYB26, more efficiently than *ARF8.2*. In T250, the intron retention of *ARF4* and *ARF8* is differentially regulated in response to drought and nitrogen starvation.

Auxin also represents a key signal for upstream regulation of the Target of Rapamycin (TOR) kinase pathway (Deng et al., 2016; Schepetilnikov et al., 2017; Pacheco et al., 2021). TOR coordinates the cell growth with nutrient or energy availability and hormonal signaling pathways (Punzo et al., 2018; Burkart and Brandizzi, 2021). In mammals, mTOR undergoes alternative splicing (Panasyuk et al., 2009). We detected IR events in TOR in T250 root, suggesting that this regulation may be present also in plants. Interestingly, in maize root the increased auxin transport and accumulation under low nitrogen condition induce root growth through TOR activation (Sun et al., 2020). Recent report showed that TOR controls alternative splicing in root (Riegler et al., 2021). We detected splicing variants of tomato homologs of TOR-associated protein LST8-2 in T250 root.

Altogether, this study has sought to identify tomato genotypes with contrasting morphophysiological behaviors under combined water and nutrient deficit and establish their expression and splicing responses to the stress applied. A number of differentially expressed genes and splice variants were identified which may contribute to describe the observed phenotypes. Further studies pursuing a detailed characterization of selected genes and alternative splicing events identified here will assess their roles and possible applications in low input agriculture.

## Data availability statement

The data presented in the study are deposited in the National Center for Biotechnology Information Sequence Read Archive, accession number PRJNA855575.

## Author contributions

AR, PP, MVO, VC, AC, and GB performed experiments; AR, PP, VC, and SE analyzed data; AM, SG, and GB directed research; GB conceived the project; GB, PP, AR, and SE wrote the manuscript with contributions from all authors. All authors contributed to the article and approved the submitted version.

## Funding

This work has received funding from the European Union’s Horizon 2020 research and innovation program under grant agreement No 727929 (A novel and integrated approach to increase multiple and combined stress tolerance in plants using tomato as a model—TOMRES), and by the CNR Bio-Memory project.

## Acknowledgments

The authors are grateful to the Department of Agriculture (IPA Sector) of the Campania Region, Prof. Amalia Barone (University of Naples Federico II), and La Semiorto Sementi srl for providing seeds. We thank Vincenzo Cenvinzo (University of Naples Federico II), Gaetano Guarino and Rosario Nocerino (CNR-IBBR) for excellent technical assistance, and Rosaria Avino and Marina Reccia for collection of experimental records.

## Conflict of interest

The authors declare that the research was conducted in the absence of any commercial or financial relationships that could be construed as a potential conflict of interest.

## Publisher’s note

All claims expressed in this article are solely those of the authors and do not necessarily represent those of their affiliated organizations, or those of the publisher, the editors and the reviewers. Any product that may be evaluated in this article, or claim that may be made by its manufacturer, is not guaranteed or endorsed by the publisher.
