# Supplementary material for: Transcriptomic and splicing changes underlying tomato responses to combined water and nutrient stress
Source: Front Plant Sci. 2022 Nov 25;13:974048. doi: 10.3389/fpls.2022.974048 (PMC9732681; doi:10.3389/fpls.2022.974048)
Supplement: Supplementary file 1 [file Presentation_1.pdf]

Materials and Methods

RNA extraction, cDNA Synthesis, and RT-qPCR

Total RNA was isolated from 100 mg of root and leaf samples using RNeasy Plant Mini Kit (Qiagen, Germany) according to manufacturer's instructions. RT-qPCR was performed as described in Punzo et al., 2020 b. Complementary DNA (cDNA) was synthesized using the QuantiTect Reverse Transcription Kit (Qiagen) from 1 µg of DNase-treated RNA. 4.5 µL of diluted (1:20) cDNA was used with 6.25 µL of Platinum SYBR Green qPCR SuperMix (ThermoFisher, USA) and 1.75 µL of primer mix (5 µM). PCR was performed using ABI 7900HT (Applied Biosystems, USA). Cycling conditions were: 10 min at 95°C followed by 40 cycles of 95°C for 15 s and 60°C for 1 min. Three biological replicates, each with three technical replicates, were tested. The relative quantification of gene expression was calculated based on the $2^{-\Delta\Delta}$Ct method (Livak and Schmittgen, 2001). Elongation factor EF1α was used as endogenous reference gene for expression normalization and RNA isolated from control plants as calibrator. Primers used are listed in Supplementary Datasheet S2

Livak, K. J., Schmittgen, T. D. (2001). Analysis of relative gene expression data using real-time quantitative PCR and the $2^{-\Delta\Delta}$CT method. Methods 25, 402–408.

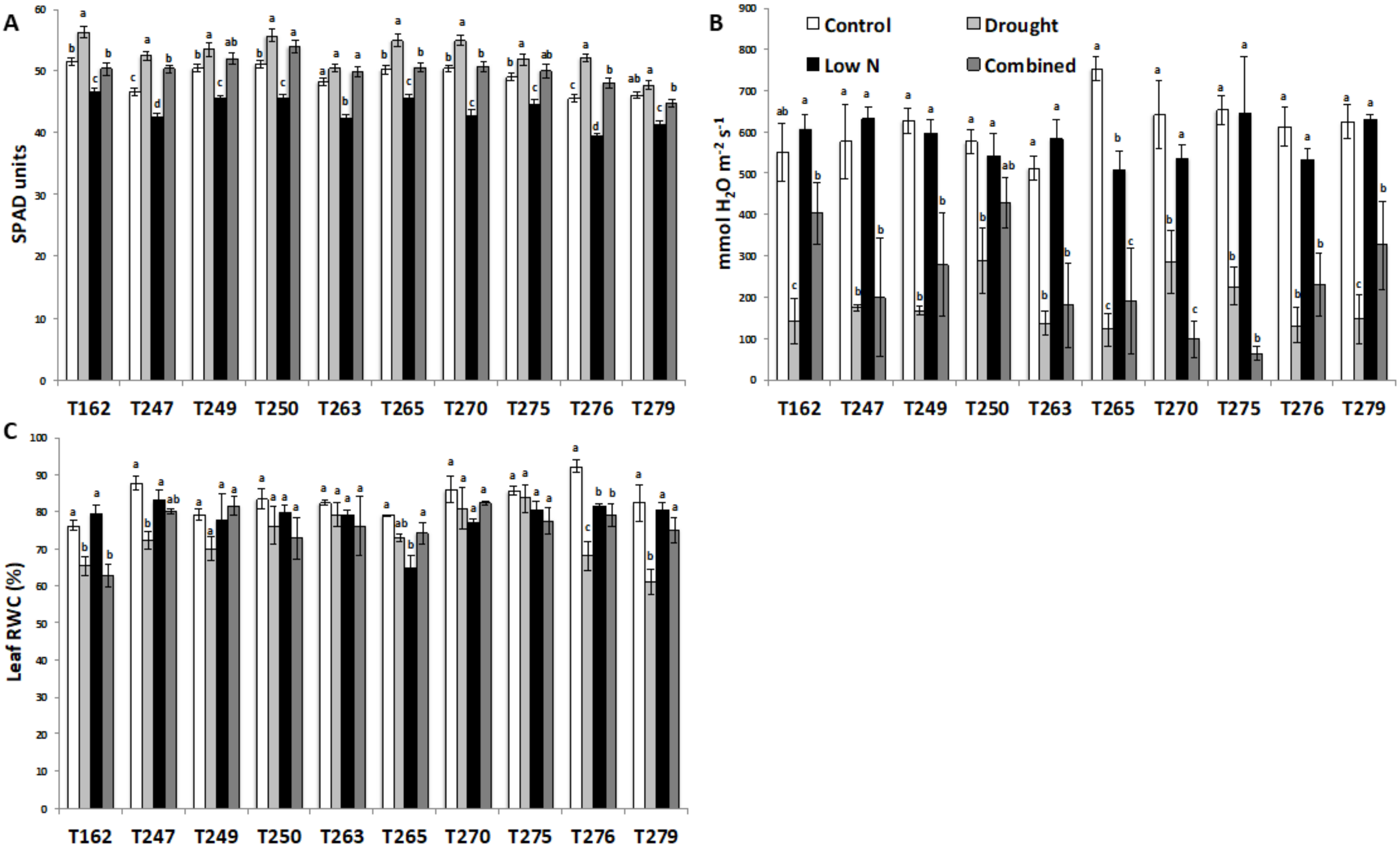

Figure S1: Physiological parameters measured after 24 days of stress in the four treatments in all genotypes. (A) Mean chlorophyll meter readings, values indicate mean ± SE (n=8); (B) Stomatal conductance; (C) Leaf relative water content. B-C, Values indicate mean ± SE (n=3). Different letters indicate significant difference within each genotype at $p < 0.05$ (Duncan test).

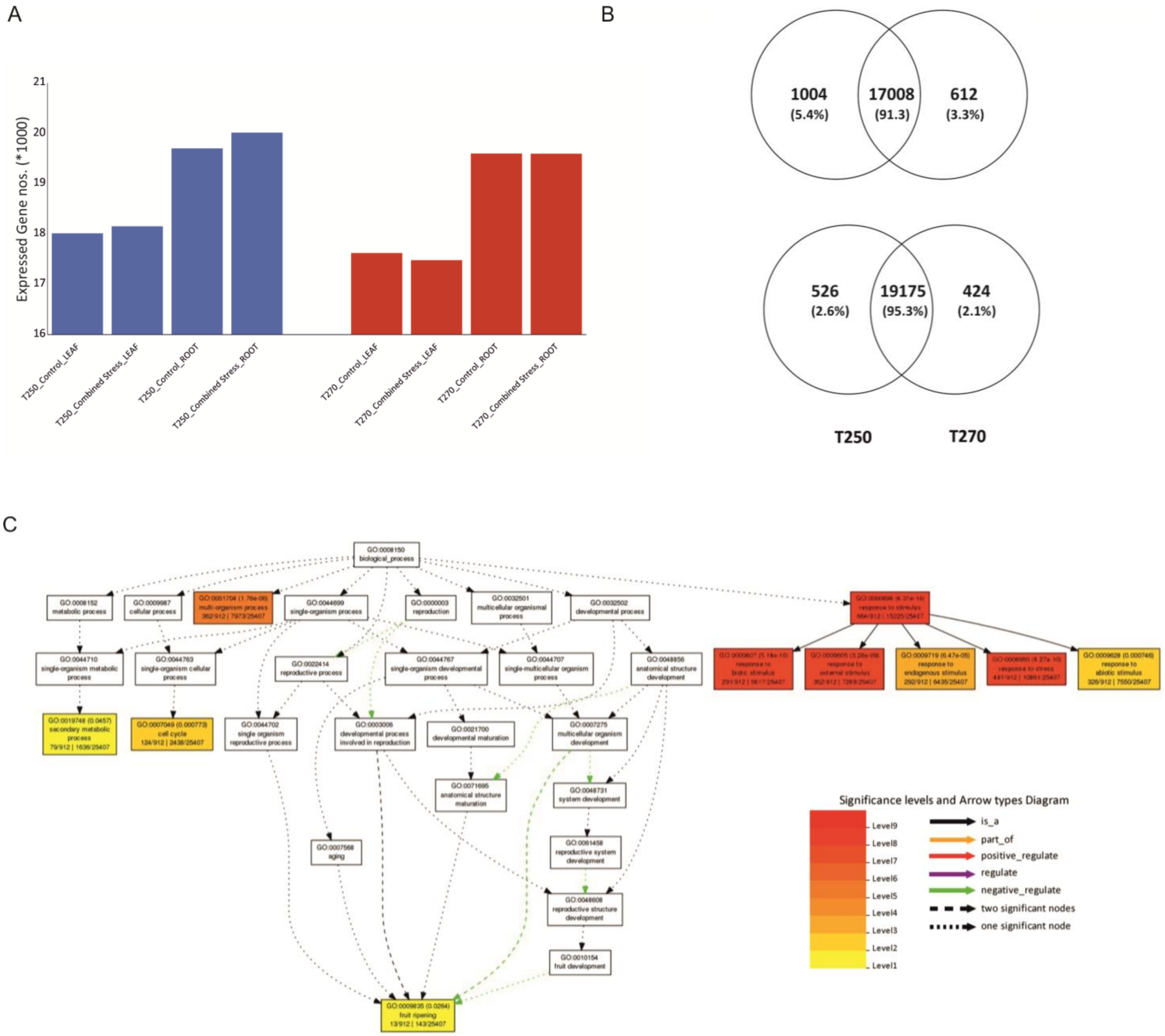

Figure S2: Expressed and Differentially Expressed Genes in roots and leaves of T250 and T270. A) Number of expressed genes (FPKM > 1) in T250 (blue bars) and T270 (red bars). Tissues and treatments are indicated; B) Venn diagrams depicting the number of genotype-specific and commonly expressed genes in leaf (upper panel) and roots (lower panel). The diagrams were drawn using the online tool Venny (Oliveros, 2007-2015); C) Gene Ontology enrichment analysis of differentially expressed genes in leaves of T250 *vs.* T270. Colors indicate significance levels, whereas different arrows represent the relationship between categories. The analysis was done using AgriGO2.0 (http://systemsbiology.cau.edu.cn/agriGOv2/).

A

locus	event	tissue	genotype	treatment	RNAseq (IncLevelDiff)	event coordinates		Skipping form RT-qPCR (RQ)	Skipping form SD	Inclusion form RT-qPCR (RQ)	Inclusion form SD
Solyc03g121880	IR	root	T270	stress	0.600	64349618	64350428	0.737	±0.017	10.364	±0.001
Solyc03g121880	IR	root	T250	stress	0.460	64349618	64350428	4.912	±0.014	6.273	±0.003
Solyc12g014280	ES	root	T270	stress	0.397	5135562	5135935	0.380	±0.001	1.748	±0.002
Solyc12g014280	ES	root	T250	stress	0.105	5135562	5135935	1.136	±0.002	1.699	±0.001
Solyc09g098330	IR	leaf	T250	stress	-0.320	68231060	68232341	3.456	±0.004	0.518	±0.001
Solyc06g076670	ES	leaf	T250/T270	control	0.305	45294222	45294333	1.019	±0.004	3.429	±0.001
Solyc01g106770	IR	root	T250/T270	control	0.681	86838150	86838727	0.341	±0.001	1.888	±0.001

B

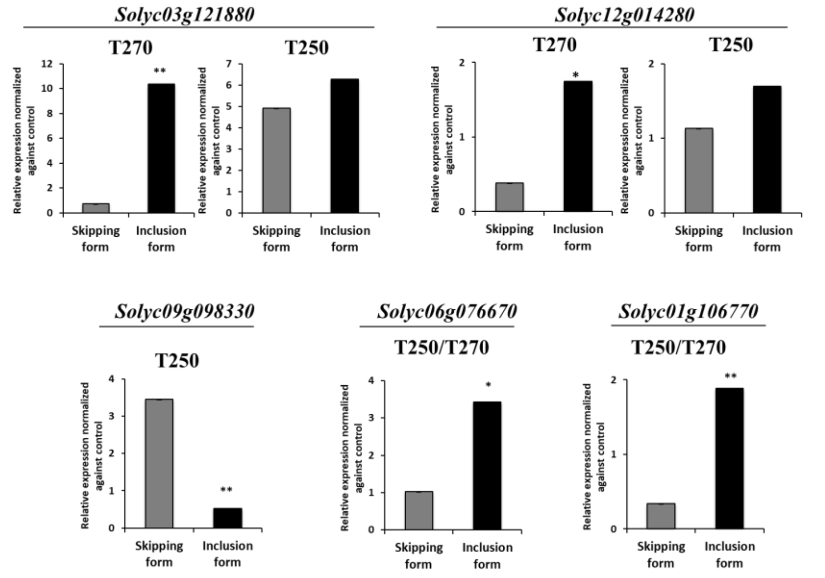

C

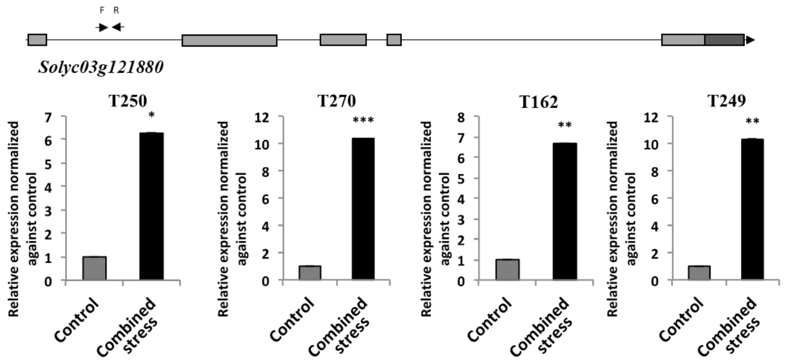

D

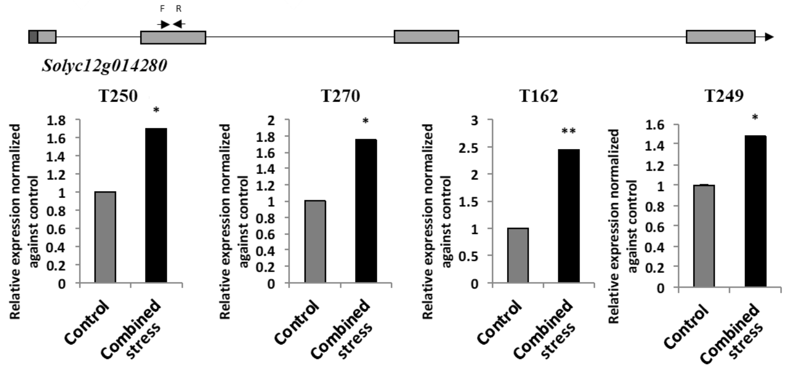

Figure S3. Validation of RNA-seq data by RT-qPCR. **A**) AS differences detected by RNA-seq were compared to the fold change obtained by RT-qPCR. **B**) Relative quantification (RQ) of expression of two variants per gene (Inclusion form and Skipping form). ES (*Solyc12g014280*, **C**) and IR (*Solyc03g121880*, **D**) events were analyzed in root of T250, T270, T162 and T249. Primers used for RT-qPCR are indicated. RNA from control plants was used as calibrator sample. The elongation factor *EF1a* was used as endogenous control. Data reported are means of three biological replicates. (*p ≤ 0.05, **p ≤ 0.01, and ***p ≤ 0.001, Student's t-test).

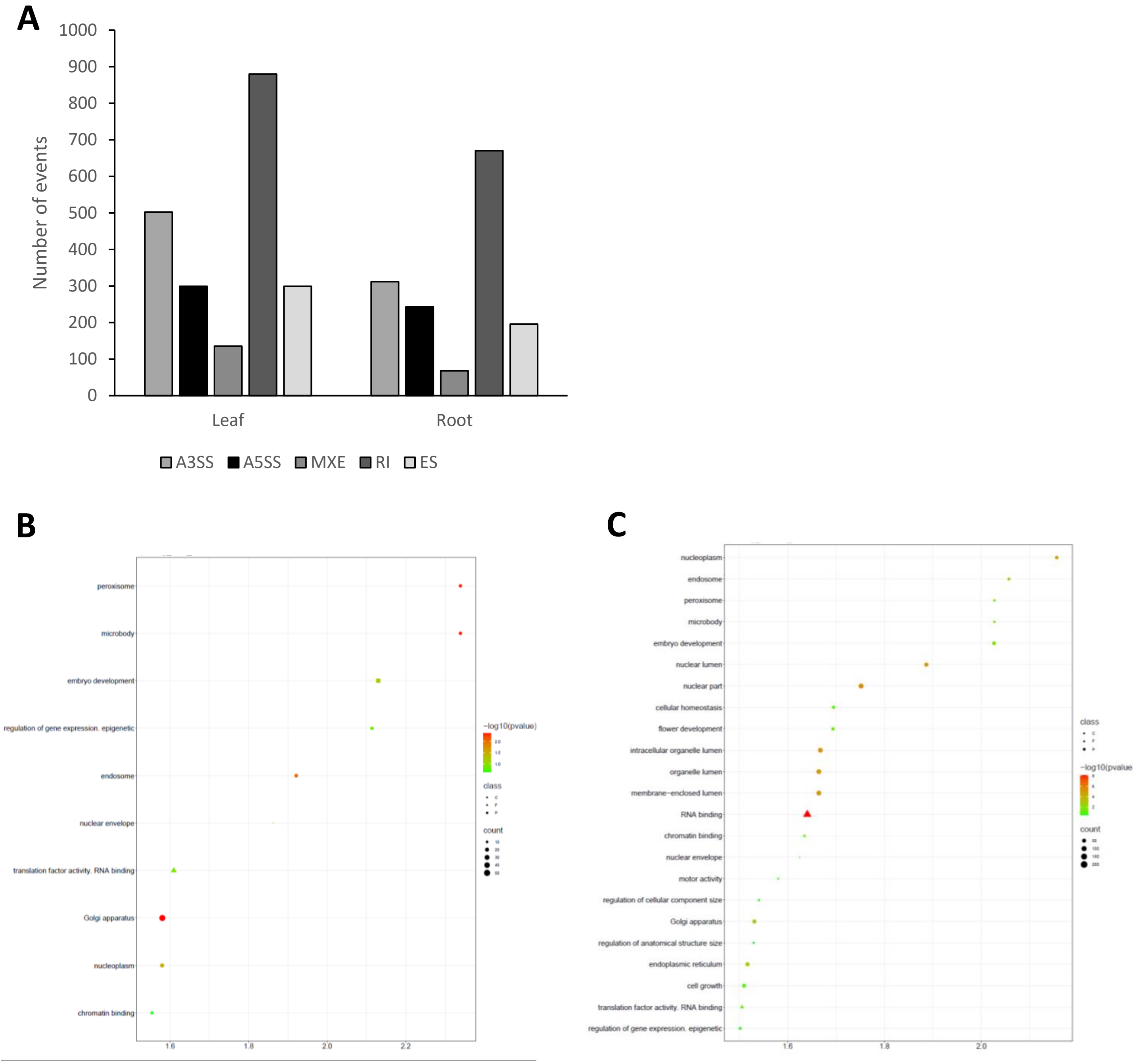

Figure S4: Alternative splicing (AS) regulation in leaf and root of T250 compared to T270 in control condition **A**) Bar graph of the number of different AS events detected in leaves and roots of T250 compared to T270. **B-C**) Gene Ontology enrichment analysis of genes undergoing differential alternative splicing in root (B) and leaf (C). Symbols indicate GO categories: MF, Molecular Function; CC, cellular compartment, BP, biological process. Symbol sizes are proportional to the gene count, whereas colors represent FDR values < 0.05. X-axis: enrichment score (ES). Only categories with ES > 1.5 and gene count > 4 are shown.

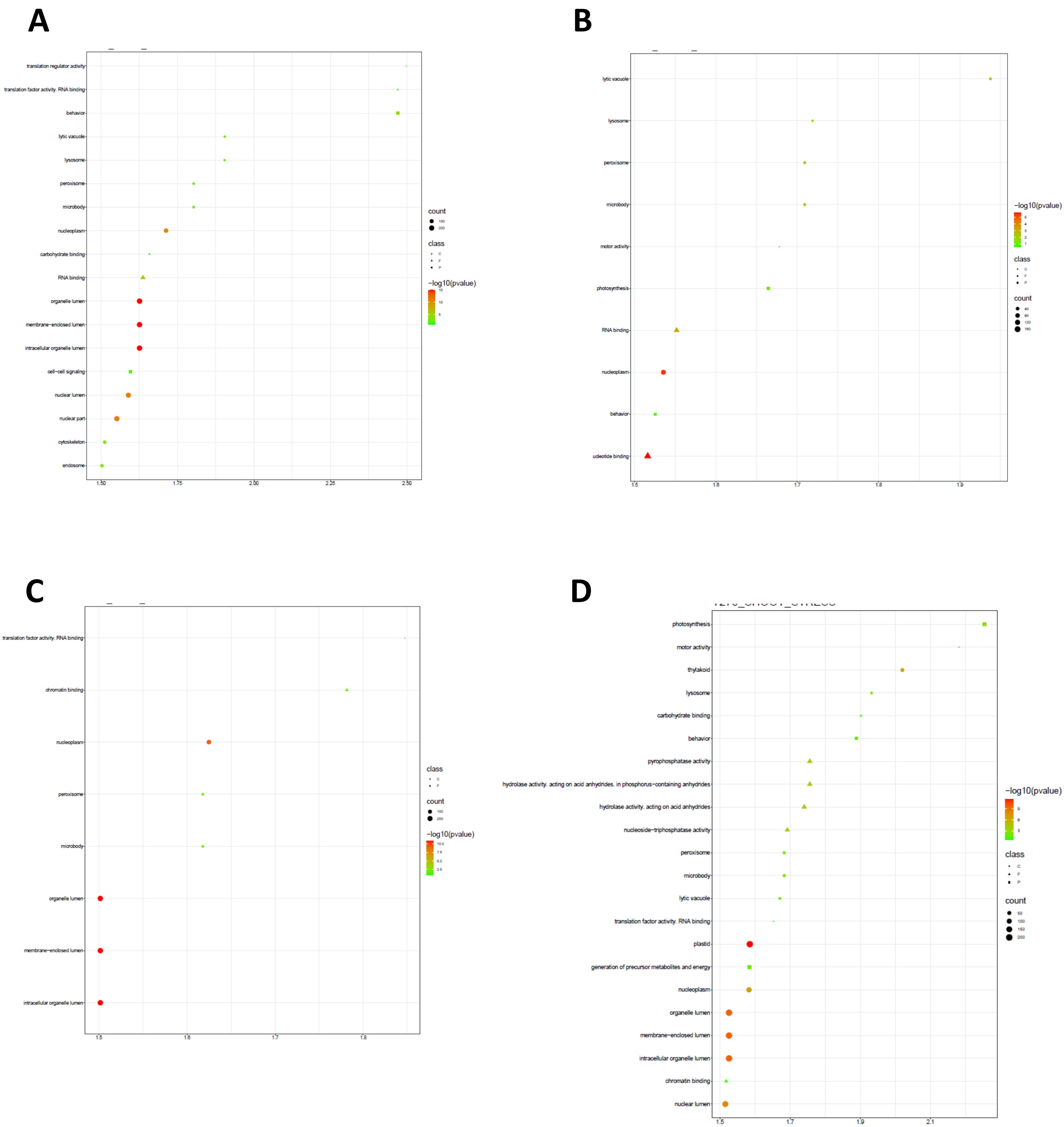

Figure S5: GO enrichment analysis of alternative splicing events in roots and leaves of T250 and T270 under combined stress condition. Genotype-specific events in T250 root (**A**) T250 leaf (**B**) T270 root (**C**) and T270 leaf (**D**) were analyzed. Symbols indicate GO categories: MF, Molecular Function; CC, cellular compartment, BP, biological process. Symbol sizes are proportional to the gene count, whereas colors represent FDR values < 0.05. Enrichment score (ES) is reported on the X-axis. Categories with ES > 1.5 and gene count > 4 are shown.
